# Direct accumulation pathway of radioactive cesium to fruit-bodies of edible mushroom from contaminated wood logs

**DOI:** 10.1038/srep29866

**Published:** 2016-07-19

**Authors:** Toshihiko Ohnuki, Yukitoshi Aiba, Fuminori Sakamoto, Naofumi Kozai, Tadafumi Niizato, Yoshito Sasaki

**Affiliations:** 1 Advanced Science Research Center, Japan Atomic Energy Agency, 2-4, Shirakata, Tokai-mura, Ibaraki, 319-1195, Japan; 2 Fujishukin Co. Ltd. 499-1 Arino Minami, Alps-city, Yamanashi, 400-0226, Japan; 3 Fukushima Environmental Safety Center, Section of Fukushima Research and Development, Japan Atomic Energy Agency, 10-2 Fukasaku, Miharu-machi, Fukushima 963-7700, Japan

## Abstract

This paper presents the accumulation process of radioactive Cs in edible mushrooms. We here first report the direct accumulation pathway of radioactive Cs from contaminated wood logs to the fruit-bodies of shiitake mushrooms through the basal portion of the stipe. In this pathway, radioactive Cs is not transported through the hyphae. This pathway results in a high accumulation of radioactive Cs in the fruit-body, more by the excess accumulation of radioactive Cs from the wood logs than that through the hyphae. We grew the fruit-bodies of Shiitake mushroom from radioactive-Cs-contaminated wood logs. The spatial distributions of radioactive Cs and Prussian blue as a tracer of interstitial water in the cross section of the wood log measured after the harvest of the fruit-body from the inoculated sawdust spawn area indicated that some fraction of the radioactive Cs and Prussian blue were transported directly to the basal portion of the stipe during the growth of the fruit-bodies.

Edible mushrooms are well known to accumulate radioactive cesium (Cs) from contaminated wood, litter, and soil^1^,^2^,^3^,^4^,^5^,^6^,^7^. Many reports have described the high accumulations of radioactive Cs in wild mushrooms collected around Europe after the Chernobyl nuclear accident^3^,^4^, and in Japan before^1^,^7^ and after^8^ the Fukushima Daiichi Nuclear Power Plant Accident. Transfer factors of radioactive Cs from substrate to wild mushroom were reported as 5.5–13^1^, 15^9^, and 9.3^10^. Even though concentration of radioactive Cs in the substrate was unique, the concentration of radioactive Cs accumulated in the wild mushroom were distributed in several orders^1^,^4^,^10^. These studies show that the accumulation of radioactive Cs by the mushroom depends on the species of the filamentous fungi.

Contamination of edible mushrooms alone was estimated to result in the high internal exposure of 4800 Bq·kg^−1^ to Fukushima residents by direct intake and/or through the food chain^11^. Some wild edible mushrooms contain higher concentration of radioactive Cs than the Japanese standard limit for general foods of 100 Bq·kg^−1^. In 2011, the investigation of dietary exposure to ^137^Cs and ^134^Cs showed that a significantly higher dose level is estimated for the residents in Fukushima than in the Kanto region and western Japan due to the intake of mushroom and fruits^12^. These results clearly showed the important effect of edible mushrooms on the internal exposure of residents after nuclear accidents. Although all kinds of mushrooms accumulate radioactive Cs, the mechanisms by which radioactive Cs accumulates in the mushroom fruit body from contaminated wood, litter, and soil have not been fully clarified.

In this report, we grew the fruit-bodies of Shiitake mushroom from radioactive-Cs-contaminated wood logs to reveal the direct accumulation pathway of radioactive Cs from contaminated wood logs to the fruit-bodies of shiitake mushrooms through the basal portion of the stipe. The spatial distributions of radioactive Cs and Prussian blue as a tracer of interstitial water in the cross section of the wood log measured after the harvest of the fruit-body from the inoculated sawdust spawn area by using autoradiography analysis and micro X-ray computed tomography system, respectively.

## Methods

### Harvest of shiitake mushroom from the contaminated wood logs

The spawn of Shiitake mushroom was prepared in grain powder mixed with and without mineral powder. Formed sawdust spawn in 12.5 mm in diameters and 20 mm hight were inoculated and covered with wax seal in the radioactive-Cs-contaminated wood logs (150 Bq·kg^−1^ ± 20 Bq·kg^−1 134^Cs + ^137^Cs). The inoculated wood logs were installed in an uncontaminated forest in Yamanashi, Japan, approximately 300 km from Fukushima Daiichi Nuclear Power Plant, for approximately 5 months. The harvested fruit-bodies collected from the wood logs were powdered for the measurement of radioactivity by an NaI(Tl) scintillation system (EMF211, EMF Japan). After the harvest of Shiitake mushrooms, the wood logs were cut to obtain a cross section at the regions where the fruit-bodies were harvested. The cut wood logs were laid on the imaging plate to obtain two-dimensional images of radioactive Cs in the wood logs by an autoradiography technique.

### Formation of fruit-body of *P*. *cystidiosus* Miller from the contaminated sawdust nutrient beds

The spawn of *P. cystidiosus* Miller were inoculated in contaminated sawdust nutrient beds containing approximately 390 Bq·kg^−1^ ± 26 Bq·kg^−1^ of ^134^Cs + ^137^Cs. *P. cystidiosus* Miller was used because of higher harvest weight than Shiitake mushroom. The sawdust nutrient bed contained no minerals, but mineral powder of a mixture of 0.2% weight vermiculite and 0.6% weight zeolite was added. The inoculated bed was kept for approximately 3 months at 20 °C until the hyphae were well colonized in the beds. The well colonized hyphae beds were moved to a temperature- and humidity-controlled room for growth of the fruit-body. The fruit bodies grown were sampled from 7 nutrient beds and merged after drying and grinding treatment to measure the radioactive Cs accumulated by an NaI(Tl) scintillation system (EMF211, EMF Japan).

### Submersion treatment of well colonized wood logs into Prussian blue dyed water

The Prussian blue, which is usually used for the accumulation of radioactive Cs from the contaminated water, dyed water was prepared by adding Prussian blue powder at 0.2% weight into water. Well colonized wood logs with no radioactive Cs contamination were submerged into the Prussian blue-dyed water at 15 °C. The Prussian blue-dyed water was introduced into the wood logs by vacuum pumping for 2 min. The submerged wood logs were placed in a temperature- and humidity-controlled room until the fruit-bodies were grown. The regions of the wood logs where the fruit-bodies had developed were cut into 13.8 × 9.6 × 14.9 mm pieces to measure the distribution of Prussian blue by micro X-ray computed tomography system (Y.CT Compact 320, YXLON).

### Growth of hyphae on agar medium containing Prussian blue dye

Separate experiments using agar medium containing nutrients, Prussian blue dye at 0.2% weight and and ^137^Cs of 46 Bq g^−1^ were conducted to test the accumulation of Prussian blue dye and ^137^Cs in the hyphae. The spawn was inoculated on the weighed membrane filter of 0.2 μm placed on agar medium. The developed hyphae on the filter were sampled together with the filter. The radioactivity of the hyphae was measured by autoradiography analysis using an imaging plate. The radioactivity in the hyphae grown on the agar medium containing ^137^Cs without Prussian blue dye was 1.6 × 10^3^ Bq g^−1^.

### Autoradiography (AR) analysis

In the AR analysis, the intensity of the imaging plate (IP) was measured by a bio imaging analyzer BAS 2500 system (Fuji Film, Japan). In order to test the measurement of radioactivity by AR analysis, we examined the relationship between the radioactivity of the standard ^137^Cs solution and the intensity of the IP exposure to the standard ^137^Cs solution dropped on the membrane filter.

## Results and Discussion

We inoculated sawdust spawn of shiitake mushrooms (*Lentinula edodes* [Berk.] Pegler) into radioactive-Cs-contaminated wood logs. The sawdust spawn contained Cs-sorbing minerals^13^,^14^ of either vermiculite or zeolite powders, each at 5% or 10% volume ratio. The concentration of radioactive Cs (^134^Cs + ^137^Cs) in the samples are shown in Table 1. Without such minerals in the spawn, the resulting fruit-body contained a similar radioactive Cs concentration to that in the wood log. The concentration of radioactive Cs in the fruit-body with the vermiculite powders of 5% or 10% in weight, or the zeolite powder of 5% or 10% in weight were approximately 80%, 60%, 80%, or 50%, respectively, of that in the fruit-body without minerals in the spawn.

**Figure 1:**
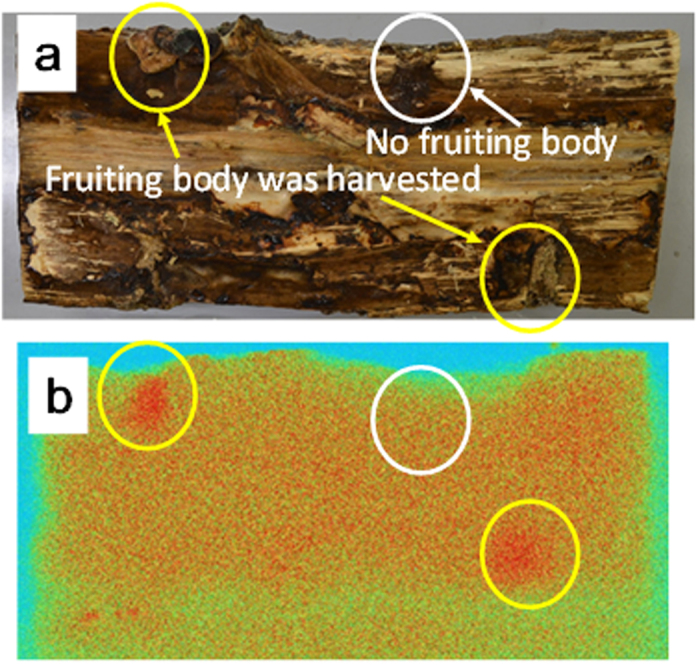
A photograph (**a**) and AR image (**b**) of the cross section of the wood log after the harvest of the fruit-bodies of shiitake mushroom. Yellow and white circles show the areas, respectively, where a fruit-body arose and did not arise from the inoculated spawn medium containing 10% weight vermiculite.

The photograph (Fig. 1a) and AR image (Fig. 1b) of the cross section of the wood log after harvest of the fruit bodies of Shiitake mushrooms (Supplementary Figure S1) showed dense areas of radioactive Cs at positions near the surface of the wood log. The dense areas circled in yellow in the AR image correspond to the inoculated spawn areas where the fruit-bodies were grown, indicating that radioactive Cs was accumulated around the fruit-body area. On the contrary, the white circle in the AR image showed that no dense spots appeared without the presence of a fruit-body even despite the presence of vermiculite powders of 10% in weight, indicating no specific accumulation of radioactive Cs in the inoculated spawn area without the formation of a fruit-body.

**Figure 2:**
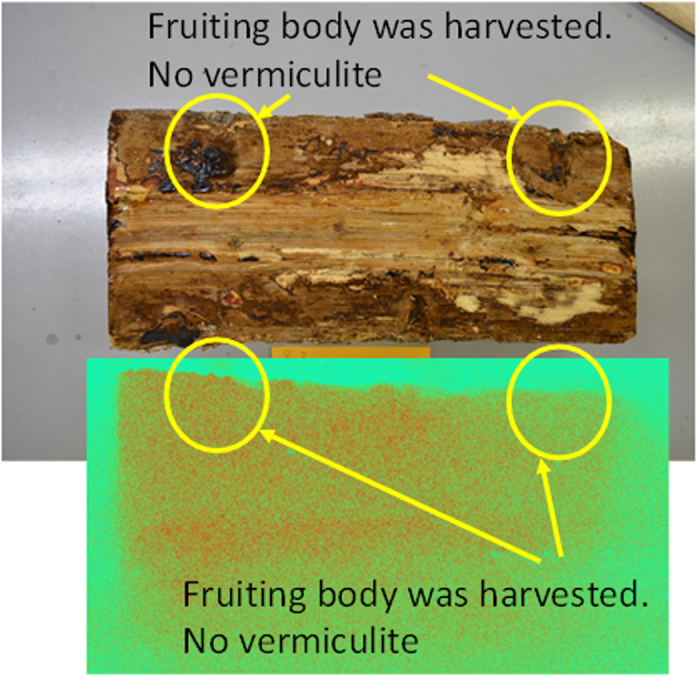
A photograph (upper image) and AR image (Lower image) of cross section of the wood log after the fruit-bodies of shiitake mushroom were harvested from the inoculated spawn medium without vermiculite.

When the sawdust spawn contained neither vermiculite nor zeolite, no specific accumulation of radioactive Cs was observed in the AR image (Fig. 2), even though the hyphae grew in the contaminated wood log irrespective of the presence of vermiculite or zeolite. These results reveal that a fraction of the radioactive Cs was intercepted by the vermiculite and zeolite during the transport of Cs from the wood log to the fruit-body at growth duration.

When we grew fruit-bodies of *Pleurotus cystidiosus* Miller in a sawdust nutrient bed containing approximately 390 Bq·kg^−1^ ± 26 Bq·kg^−1^ of ^134^Cs + ^137^Cs, the fruit-body contained 71 Bq·kg^−1^ of ^134^Cs and ^137^Cs. Addition of the mineral powder to the sawdust nutrient bed lowered the concentration of radioactive Cs in the fruit-body to 9.6 Bq·kg^−1^ ±  1.6 Bq·kg^−1^ of ^134^Cs and ^137^Cs. This result indicates that presence of the mineral mixture in the whole region of the medium reduced the concentration of radioactive Cs in the fruit-body more effectively than in the wood log experiment, where minerals were present only in the spawn plugs. The mineral powders do not sorb radioactive Cs directly from the sawdust powder, but from the interstitial pore water. Thus, for the contaminated wood logs, radioactive Cs dissolved in the interstitial pore water was sorbed by the mineral powders.

**Figure 3:**
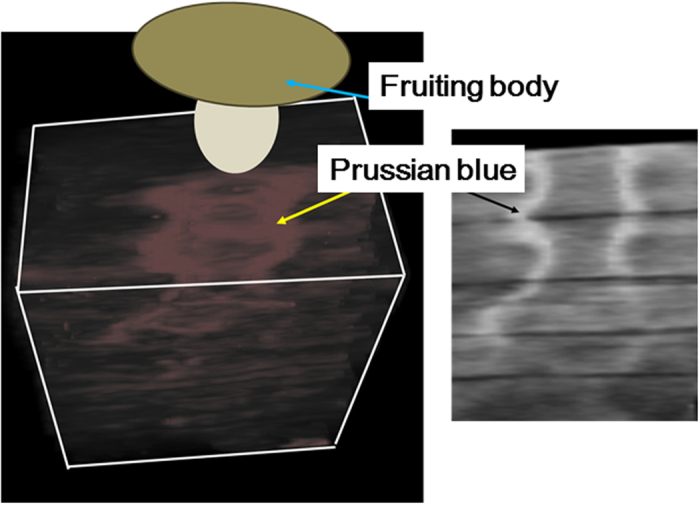
Three-dimensional distribution (**a**) and cross section of distribution of nano-sized Prussian blue after harvesting the fruit-bodies from the surface of the wood log. The three-dimensional distribution the Prussian blue in the wood log was determined by X-ray CT analysis, which detected dense materials in the materials. Prussian blue contains Fe in its structure. The determined distribution was identified as that of Prussian blue. The fruit body was illustrated as an image based on the photograph taken after the fruit body was harvested.

In growing Shiitake mushrooms, the wood logs are usually submerged in water for 1~2 days in order to stimulate the formation of the fruit-bodies from the well-colonized hyphae in wood logs. This submersion treatment dissociates radioactive Cs from the contaminated wood logs into the interstitial water. Thus, the dissolved radioactive Cs in the submerged water of the wood logs was sorbed by the minerals during transport to the fruit-body. We added nano-sized dye of Prussian blue at 10% weight to the submersion water as a tracer of this water. After harvesting the Shiitake mushrooms from a non-contaminated wood log, the distribution of the nano-sized Prussian blue was measured by micro X-ray CT analysis. The nano-sized Prussian blue was distributed just beneath the fruit-bodies (Fig. 3a). The three-dimensional distribution of the Prussian blue (Supplementary Video S1) showed that the Prussian blue powders were distributed in an ellipsoidal shape from the fruit-body. The cross section of the distribution of Prussian blue (Fig. 3b) showed the presence of an empty area in the center of the ellipse underneath the fruit-body. The color of the fruit-body was not changed (Supplementary Figure S2(a)) while the color beneath the fruit-body was changed to blue (Supplementary Figure S2(b)). These results reveal that the interstitial pore water was transported toward the fruit-body.

**Figure 4:**
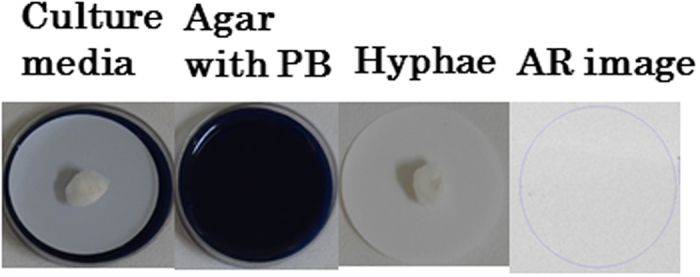
Photographs of *Marasmiaceae* sp. hyphae grown on agar medium containing 0.1wt% Prussian blue; (**a**) hyphae on the membrane filter set on the agar medium, (**b**) the agar medium after separation of the hyphae with the membrane filter, (**c**) the hyphae with the membrane filter, and (**d**) an AR image of the hyphae with the membrane filter showed that the radioactive Cs accumulated in the hyphae was below the detection limit.

The hyphae of Shiitake mushroom were grown on a membrane filter placed on agar medium containing Prussian blue at 0.1% weight (Fig. 4: Culture media), which changes color in the medium to dark blue (Fig. 4: Agar with PB). Even though the color of the medium was dark blue, the color of the hyphae was white (Fig. 4: Hyphae) and radioactivity in the hyphae was 0.18 ± 0.022 Bq g^−1^, showing that Prussian blue and ^137^Cs did not penetrate into the hyphae. This result clearly suggests that the Prussian blue which accumulated ^137^Cs in the submersion water was not transported to the hyphae, but through the interstitial water in the wood log outside of the hyphae. It is reported that the addition of the Prussian blue in submersion water of contaminated wood logs decreases the concentration of radioactive Cs in the fruit-body^15^. These findings reveal that accumulation of radioactive Cs from the wood log to the fruit-body results from two processes. One is the pathway by which the radioactive Cs was accumulated through the hyphae. The other is the pathway by which radioactive Cs is transported directly from the interstitial pore water to the fruit-body.

Radioactive Cs is highly accumulated in the fruit-bodies of filamentous fungi. Since radioactive Cs is known to accumulate in hyphae, it is believed that radioactive Cs is transported to the fruit-body through hyphae^16^. Cesium accumulated in the hyphae of *Pleurotus ostreatus* is trapped by intercellular materials of polyphosphate in vacuoles or other organs^2^. Indeed, hyphae function in the uptake and transport of the radioactive Cs dissolved in the interstitial water into the fruit-bodies^17^. Our results showed the presence of a direct pathway of radioactive Cs accumulation into the fruit-body from the contaminated wood logs. A previous study on the accumulation paths of radioactive Cs in hyphae using inhibitors of uptake through channels suggested a possible indirect pathway of Cs accumulation in fruit-bodies by extracellular transport via inter-hyphal cavities^18^. Unfortunately, that study could not show direct evidence of extracellular transport. In the forest the fruit-bodies of edible and inedible mushrooms tend to grow after rain events. The rain water dissolves radioactive Cs in the litter zone^19^. Some portion of the dissolved radioactive Cs is transported directly to the fruit-body, causing excess accumulation of radioactive Cs in the fruit-body rather than through hyphae. Therefore, direct accumulation pathway of radioactive Cs from the contaminated wood, litter, and soil should be included to understand the migration of radioactive Cs in forest.

Fungal hyphae function strongly for the detention of radioactive Cs in organic layers in the forest system^20^. The presence of minerals in agar medium inhibits the accumulation of radioactive Cs by unicellular fungus^21^. Our results showed that the presence of minerals at the position near the fruit-body decreased the concentration of radioactive Cs in the fruit-body. This result reveals that radioactive Cs is eliminated by minerals from the interstitial pore water during transport to the fruit-body. Thus, epi-scattering of the minerals of zeolite and/or vermiculite onto the litter zone of a contaminated forest can be expected to reduce the accumulation of radioactive Cs by edible and inedible mushrooms. One can assume that radioactive Cs transported through hyphae is released outside of the hyphae, and is sorbed by the minerals. The AR image of the wood log (Fig. 1) shows that no dense spot appeared without growth of the fruit-body even for the inoculated spawn containing 10% weight vermiculite powder. These results indicate that most of the radioactive Cs in the hyphae is not released from hyphae to be sorbed by minerals, but is transported into the fruit-bodies.

## Additional Information

**How to cite this article**: Ohnuki, T. *et al*. Direct accumulation pathway of radioactive cesium to fruit-bodies of edible mushroom from contaminated wood logs. *Sci. Rep.* **6**, 29866; doi: 10.1038/srep29866 (2016).

## Supplementary Material

Supplementary Information

Supplementary Video S1

## Figures and Tables

**Table 1:** Radioactivity in fruit-body of shiitake mushrooms (*Lentinula edodes* [Berk.] Pegler) and in wood logs of 12% D/W and transfer factors of radioactive Cs from the contaminated wood logs to the fruit-body.

Sample	Radioactivity (Bq kg^−1^)		Transfer factor	Standard deviation
	Wood log	Fruit-body		
Without mineral	161	163	1.01	0.17
Vermiculite 5%	145	118	0.81	0.14
Vermiculite 10%	158	98	0.62	0.17
Zeolite 5%	140	108	0.78	0.10
Zeolite 10%	172	85	0.5	0.08

## References

[b1] Yoshida S., Muramatsu Y. & Ogawa M. Radiocesium concentrations in mushrooms collected in Japan. J. Environ. Radioact. 22, 141–154 (1994).

[b2] Sugiyama H. . Accumulation and Localization of Cesium in Edible Mushroom (Pleurotus ostreatus) Mycelia. J. Agric. Food Chem. 56, 9641–9646 (2008). 18800803 10.1021/jf801269t

[b3] Heinrich G. Uptake and transfer factors of 137Cs by mushrooms. Radiat. Environ. Biophys. 31, 39–49 (1992). 1589573 10.1007/BF01211511

[b4] Kammerer L., Hiersche L. & Wirth E. Uptake of radiocesium by different species of mushrooms. J. Environ. Radioact. 23, 135–150 (1994).

[b5] Mietelski J. W., Jasin´ska M., Kubica B., Kozak K. & Macharski P. Radioactive contamination of Polish mushrooms. Science of the Total Environment 157, 217–226 (1994).

[b6] Kalac P. A review of edible mushroom radioactivity. Food Chemistry 75, 29–35 (2001).

[b7] Kuwahara C. . Accumulation of radiocesium in wild mushrooms collected from a Japanese forest and cesium uptake by microorganisms isolated from the mushroom-growing soils. Science of the Total Environment 345, 165–173 (2005). 15919537 10.1016/j.scitotenv.2004.10.022

[b8] Hamada N. & Ogino H. Food safety regulations: what we learned from the Fukushima nuclear accident. J. Environ. Radioact. 111, 83–99 (2012). 21996550 10.1016/j.jenvrad.2011.08.008

[b9] Eckl P., Hofmann W. & Turk R. Uptake of natural and man-made radionuclides by lichens and mushrooms, Radat. Environ, Biophys. 25, 43–54 (1986). 10.1007/BF01209684 3714974

[b10] Tsukada H., Shibata H. & Sugiyama H. Transfer of radiocaesium and stable caesium from substrata to mushrooms in a pine forest in Rokkasho-mura, Aomori, Japan. J. Environ. Radioact. 39, 149–160 (1998).

[b11] Tsubokura M. . Reduction of High Levels of Internal Radio-Contamination by Dietary Intervention in Residents of Areas Affected by the Fukushima Daiichi Nuclear Plant Disaster: A Case Series. PLOS ONE 9, e100302, 10.1371/journal.pone.0100302 (2014). 24932486 PMC4059713

[b12] Harada K. H. . Dietary Intake of Radiocesium in Adult Residents in Fukushima Prefecture and Neighboring Regions after the Fukushima Nuclear Power Plant Accident: 24-h Food-Duplicate Survey in December 2011. Environ. Sci. Technol. 47, 2520–2526 (2013). 23259847 10.1021/es304128t

[b13] Ohnuki T. Sorption Characteristics of Cesium on Sandy Soils and Their Components. Radiochimica Acta 65, 75–80 (1994).

[b14] Ohnuki T. & Kozai N. Adsorption behavior of radioactive cesium by non-mica minerals. J. Nucl. Sci. Technol. 50, 369–375 (2013).

[b15] Kunitomo Y. & Sakata H. Practical method for decreasing the transfer of radioactive Cs to mushrooms from wood logs by using Prussian blue. 31, Mar., http://www.pref.gunma.jp/contents/000300719.pdf (in Japanese) 2015).

[b16] Vinichuk M. M., Johanson K. J., Rosen K. & Nilsson I. Role of the fungal mycelium in the retention of radiocaesium in forest soils. J. Environ. Radioact. 78, 77–92 (2005). 15465181 10.1016/j.jenvrad.2004.02.008

[b17] Bazala M. A., Go1da K. & Bystrzejewska-Piotrowska G. Transport of radiocesium in mycelium and its translocation to fruitbodies of a saprophytic macromycete. J. Environ. Radioact. 99, 1200–1202 (2008). 18313816 10.1016/j.jenvrad.2008.01.004

[b18] Bystrzejewska-Piotrowska G. & Bazala M. A. A study of mechanisms responsible for incorporation of cesium and radiocesium into fruitbodies of king oyster mushroom (*Pleurotus eryngii*). J. Environ. Radioact. 99, 1185–1191 (2008). 18342998 10.1016/j.jenvrad.2008.01.016

[b19] Sakai M. . Radiocesium leaching from contaminated litter in forest streams. J. Environ. Radioact. 144, 15–20 (2015). 25791899 10.1016/j.jenvrad.2015.03.001

[b20] Dighton J., Clint G. M. & Poskitt J. Uptake and accumulation of Cs-137 by upland grassland soil fungi: a potential pool of Cs immobilization. Mycol. Res. 959, 1052–1056 (1991).

[b21] Ohnuki T. . Effect of minerals on accumulation of Cs by fungus *Saccaromyces cerevisiae*. J. Environ. Radioact. 144, 127–133 (2015). 25841115 10.1016/j.jenvrad.2015.02.018

