# Supplementary material for: Direct accumulation pathway of radioactive cesium to fruit-bodies of edible mushroom from contaminated wood logs
Source: Sci Rep. 2016 Jul 19;6:29866. doi: 10.1038/srep29866 (PMC4949424; doi:10.1038/srep29866)
Supplement: Supplementary Information [file srep29866-s1.pdf]

Supplementary Information for

Direct accumulation pathway of radioactive cesium to fruit bodies of edible mushroom from contaminated wood logs

Toshihiko Ohnuki[1]†, Yukitoshi Aiba[2], Fuminori Sakamoto[1], Naofumi Kozai[1], Tadafumi Niizato[3], and Yoshito Sasaki[3]

[1]Advanced Science Research Center, Japan Atomic Energy Agency, 2-4, Shirakata, Tokai-mura, Ibaraki, 319-1195, Japan
[2]Fujishukin Co. Ltd. 499-1 Arino Minami, Alps-city, Yamanashi, 400-0226, Japan
[3]Fukushima Environmental Safety Center, Section of Fukushima Research and Development, Japan Atomic Energy Agency, Sahei 8F, 1-29, Okitama-cho, Fukushima-shi, Fukushima 960-8034, Japan

To whom correspondence should be addressed.
Tel +81-29-282-5535 e-mail:ohnuki.toshihiko@jaea.go.jp

Table of contents

Supplementary Figure S1 and 2, Supplementary Video S1, and their legends

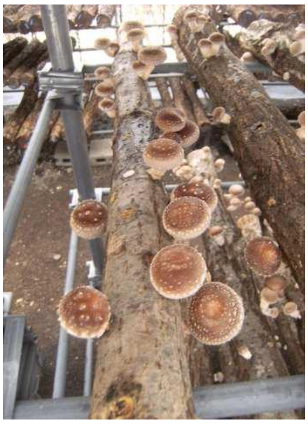

Figure S1. Fruit bodies of Shiitake mushroom grown from the contaminated wood logs.

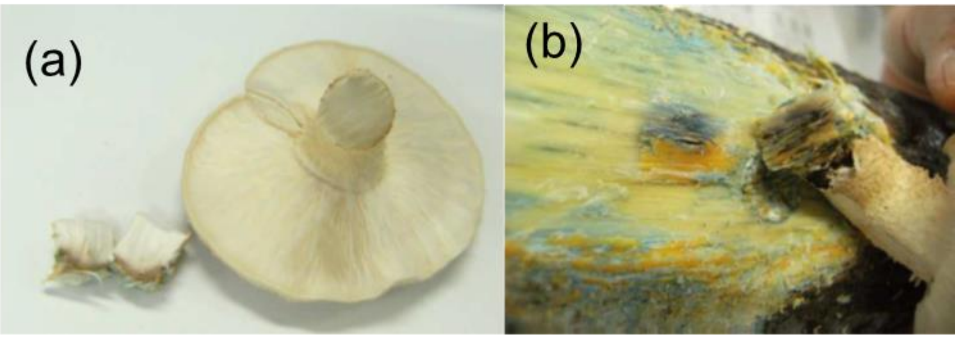

Figure S2. Fruit bodies of shiitake mushroom grown from the wood logs submerged in Prussian blue-dyed water. (a) Photograph of fruit body, (b) wood log beneath the basal portion of the fruit body. The color of fruit body was not changed, but the region in the wood logs beneath the basal portion was changed.

Supplementary Video S1

Three-dimensional distribution of Prussian blue in the region of wood log around fruit body. Colored zone showed the presence of Prussian blue.

.
